# Role of Laryngopharyngeal Reflux Changes in Children with Adenoid Hypertrophy: A Randomized Controlled Prospective Study

**DOI:** 10.1155/2023/5628551

**Published:** 2023-02-06

**Authors:** Yu Zhou, Ruixia Ma, Jiangbo Luo, Zhikai Wang, Pei Yang

**Affiliations:** ^1^Department of Otorhinolaryngology Head and Neck Surgery, General Hospital of Ningxia Medical University, Yinchuan, Ningxia 750001, China; ^2^Department of Otorhinolaryngology Head and Neck Surgery, The First People's Hospital of Yinchuan, Yinchuan, Ningxia 750001, China; ^3^Department of Otorhinolaryngology Head and Neck Surgery, Ningxia Wuzhong Hongsipu District People's Hospital, Wuzhong, Ningxia 750001, China

## Abstract

**Objectives:**

This prospective randomized controlled analysis aimed to assess the changes in laryngopharyngeal reflux (LPR) in children with adenoid hypertrophy (AH). Study design: a prospective, randomized, and controlled analysis.

**Methods:**

The reflux symptom index (RSI) and the reflux finding score (RFS) scores were used to evaluate the laryngopharyngeal reflux changes in children diagnosed with adenoid hypertrophy. The pepsin concentration in salivary samples was examined, and the positive pepsin was used to assess the sensitivity and specificity of RSI, RFS, and RSI combined with RFS in forecasting LPR.

**Results:**

In 43 children with AH, the sensitivity of the RSI and RFS scale (used alone or in combination) in diagnosing pharyngeal reflux in children with adenoid hypertrophy was lower. Pepsin expression was identified in 43 items of salivary samples, with a total positive rate of 69.77%, most of which were optimistic. The expression level of pepsin was positively correlated with the grade of adenoid hypertrophy (*r* = 0.576, *P* < 0.01). Based on the positive rate of pepsin, we found that the sensitivity and specificity of RSI and RFS were 5.77%, 35.03%, and 91.74%, 55.89%. Moreover, there was a noticeable distinction in the number of acid reflux episodes between the LPR-positive and LPR-negative groups.

**Conclusion:**

There is a special connection between LPR change and children's AH. LPR exerts a crucial role in the progression of children's AH. Because of the low sensitivity of RSI and RFS, it is not suitable for LPR children to choose AH.

## 1. Introduction

Adenoid hypertrophy (AH) is one of the most common respiratory diseases in children. The incidence rates of AH in children and adolescents range from 40 to 71% [1]. The main clinical symptoms of AH are snoring, mouth-open breathing, nasal congestion, and so on. Remarkably, it has been shown that long mouth-open breathing may lead to an “adenoid face” [2]. Adenoids are the immune organs of the body, containing lymphocytes at different stages of development [3]. Adenoid hypertrophy may block the eustachian tube's posterior nostril and pharyngeal orifice, leading to diseases of the adjacent organs [4]. One of the most critical complications of AH is obstructive sleep apnea (OSA), which has a significant impact on children's growth and learning [5]. Therefore, AH has been paid more and more attention by clinicians and parents, striving for early diagnosis and treatment.

Laryngopharyngeal reflux (LPR) is an upper respiratory tract (UAT) disease, which is related to the reflux of gastric or duodenal contents, resulting in morphological changes of UAT [6]. LPR causes damage and structural changes to laryngeal epithelial mucosa through repeated stimulation of the throat mucosa by gastric acid, pepsin, and bile acid in the gastric contents [7]. Emerging pieces of evidence suggests that 5%–10% of patients in the otolaryngology department have symptoms of LPR [8]. Recent studies have shown that LPR might be one of the pathogenic factors for adenoid hypertrophy in children [9]. A recent study studied 30 children with adenoidal hypertrophy and found that gastroesophageal reflux in infants under 1-year-old was 88% and 32% for infants under one-year-old [10]. Another group reported that the prevalence of LPR was 46.7% in 30 children with adenoidal hypertrophy, which is much higher in the control group [11]. Similarly, it has been found that the reflux substances such as gastric acid and pepsin might act as antigens to stimulate the immune response of adenoid lymphoid tissue, resulting in AH [12]. However, the study on the laryngopharyngeal reflux changes in children's adenoid hypertrophy is mainly unknown.

In this study, the expression of pepsin in adenoid tissue was detected to investigate the correlation between adenoid hypertrophy and pharyngeal reflux change. The possibility and accuracy of the RSI and RFS in predicting the correlation between children's adenoid hypertrophy and LPR change were assessed.

## 2. Materials and Methods

### 2.1. Clinical Data

Children with adenoidal hypertrophy (nasal obstruction >50%) in the department of otorhinolaryngology in the General Hospital of Ningxia Medical University were selected for the study. Inclusion criteria were as follows: Nasal endoscopy showed adenoid grade III∼IV, mouth-open breathing, snoring, recurrent or chronic otitis media, or sinusitis caused by adenoid hypertrophy. Exclusion criteria were follows: (1) Antacid drugs that have been used in the recent three months; (2) suffering from immune system diseases or other severe system diseases; (3) upper respiratory tract infection occurred within two weeks; and (4) long-term use of antibiotics, hormones, and other drugs in the recent three months. This study was approved by the Ethics Committee of the General Hospital of Ningxia Medical University. All experimental samples were obtained and used with the written consent of the patient's family.

### 2.2. Electronic Nasopharyngoscopy

All children underwent electronic nasopharyngoscopy before the operation, and adenoid hypertrophy was divided into four grades according to the grading method proposed by Cassano and Gelardi [13]. Level I: nasal cavity ≤25% after hypertrophic adenoid obstruction; Level II: 26%–50% of nostrils after hypertrophic adenoids block; Level III: 51%–75% of nostrils after hypertrophic adenoids block; and Level IV: 76%–100% of nostrils after hypertrophic adenoids block. Hypertrophy of grade III ∼ IV with clinical symptoms is adenoidal hypertrophy.

### 2.3. RFS and RSI Scores

The RSI score developed by Belafsky was filled in by the family members (young children) or children (older children). Each item was divided into 0–5 points according to the throat symptoms, and the total score >13 points were positive; otherwise, it was negative. Two doctors independently scored the physical signs under the electronic laryngoscope. According to the RFS scale developed by Belafsky, the total score >7 was positive; otherwise, it was negative.

### 2.4. Detection of Pepsin Concentration in Saliva

Saliva was collected from the patients who participated in the study. Patients were offered 30 mL of germ-free pliable pipes to collect saliva. Pliable pipes were centrifuged for 10 min at 5000 rpm, collected the supernatant, and added to the human pepsin kit (Yonghui Biotechnology Co., Ltd.). Salivary samples were examined using an automatic enzyme labeling instrument (Thermo). Sixteen ng/ml was used as the critical value to consider the positive pepsin of salivary samples.

### 2.5. Statistical Analysis

SPSS 22.0 software was used for data processing. Continuous data were expressed as mean ± standard deviation if normally distributed and as median or else. A *T*-test is used for normal distribution and homogeneity of variance, and the Wilcoxon test is used for otherwise. Spearman correlation analysis is selected for nonbivariate normal distribution data. The statistically significant value is *P* < 0.05.

## 3. Results

### 3.1. RSI Scores in Children with Adenoidal Hypertrophy

Among 43 children with AH, 21 cases (48.8%) had grade III hypertrophy, and 22 cases (51.2%) had grade IV hypertrophy. Among them, 2 cases (4.7%) had RSI >13 points, 14 cases (32.6%) had RFS >7 points, and 1 case was positive in both scores. According to the RSI scores, the symptoms of 43 children with AH included: hoarseness or dysphonia in 5 cases (11.6%), excessive sputum or nasal regurgitation in 24 cases (55.8%), cough after eating or lying down in 17 cases (35.9%), throat clearing in 25 cases (58.1%), dyspnea in 4 cases (9.3%), annoying cough in 23 cases (53.5%), heartburn, chest pain, and stomach pain in 2 cases (4.7%, Table 1).

### 3.2. RFS Scores in Children with Adenoidal Hypertrophy

According to the RFS scores, 43 children with adenoidal hypertrophy showed symptoms under the electronic laryngoscope: 5 cases (11.6%) of pseudovocal fold groove, 10 cases (23.3%) of partial or complete disappearance of the laryngeal chamber, 6 cases (13.9%) of diffuse erythema of laryngeal cavity, 12 cases (27.9%) of vocal cord edema, 23 cases (53.5%) of mucus attachment in the larynx, 6 cases (13.9%) of in diffuse laryngeal edema, 7 cases (16.3%) of in thickening of posterior commissural mucosa, and 0 cases (0%) of granuloma (Table 2).

### 3.3. Correlation Analysis of Adenoidal Hypertrophy and Pepsin Expression

**Figure 1:**
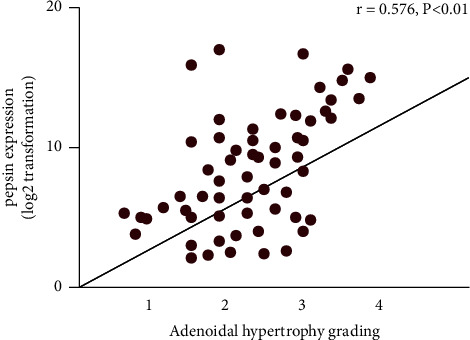
The relationship between AH grading and pepsin expression.

Pepsin, a reliable marker for diagnosing laryngeal reflux, has been abundantly recognized in various diseases, such as vocal cord polyps in adults and laryngeal malacia in children [12]. In this study, the expression of pepsin in salivary samples was detected. Based on the thresholds of 16 ng/mL, the prevalence of positive pepsin was markedly increased in LPR (+) group compared with that in the LPR (−) group (*P* < 0.05). Furthermore, the pepsin concentration was markedly enhanced in the LPR (+) group compared with that in the LPR (−) group (*P* < 0.05). In 43 cases of salivary samples, the total positive rate of pepsin is 69.77%, of which 7 cases (16.28%) were strongly positive, 13 cases (30.23%) were positive, 9 cases (20.93%) were weakly positive, and 13 cases (30.23%) were negative. Spearman's correlation analysis showed that the higher the grade of AH, the higher the pepsin expression intensity (Figure 1, Table 3; *r* = 0.576, *P* < 0.01).

### 3.4. Consistency Analysis between Pepsin Expression in Adenoids and Reflux Tables

The positive pepsin in adenoid tissues was used as the diagnostic standard of pharyngeal reflux. Based on the pepsin expression, the sensitivity of RSI, RFS, and RSI combined with RFS was 5.77%, 35.03%, and 38.46%, respectively, and the specificity was 91.74%, 55.89%, and 53.67%, respectively (Table 4).

### 3.5. Patients' Characteristics

The characteristics of patients with LPR diagnosis are exhibited in Table 5. A total of 43 children with adenoidal hypertrophy were enrolled in the study. The age range of the children was 2–10 years old, with an average of 5.4 years old. The course of the disease was 3–52 months, with an average of 16.3 months. LPR was detected in 8 (18.6%) subjects. No differences in age, sex, LPR, acid regurgitation, mouth breathing, snoring, stoppage of breathing, URTI, and heartburn were observed between patients in the LPR (+) and LPR (−) groups.

### 3.6. The Reflux Episodes Number in LPR Positive and Negative Patients

**Figure 2:**
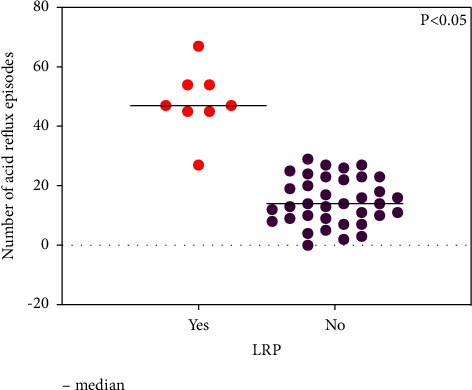
Number of acid reflux episodes in LPR (+) and LPR (−) groups.

**Figure 3:**
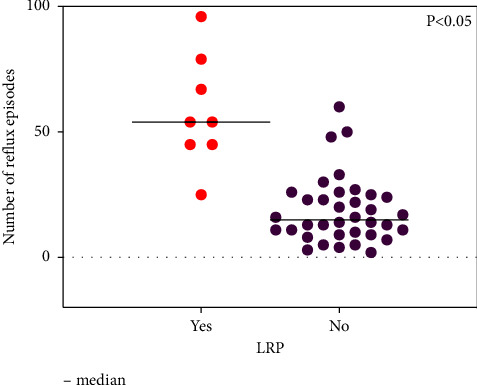
Total quantity of reflux episodes in LPR (+) and LPR (−) groups.

Figures 2 and 3 show a marked difference in the number of acids and total quantity of reflux episodes between the LPR-positive and LPR-negative groups (all *p* < 0.05).

## 4. Discussion

Adenoidal hypertrophy often causes nasal congestion and mouth-opening breathing in children and affects physical development [14]. Increasing evidence indicates that AH might be caused by viruses, bacterial infections, allergic inflammation, the influence of second-hand smoke, and other factors [15]. In recent years, studies have found that throat reflux plays a critical role in the occurrence and development of adenoid hypertrophy, chronic sinusitis, chronic rhinitis, secretory otitis media, asthma, and laryngeal dysfunction. Keles et al. performed 24-hour esophageal pH monitoring in 30 children with adenoidal hypertrophy and observed that the incidence of pharyngeal reflux was significantly enhanced compared with that in the control group [16]. They indicated that pharyngeal reflux plays an essential role in the process of adenoidal hypertrophy. A recent study investigated 207 children diagnosed with chronic adenoiditis and observed that 87 cases were suspected of upper respiratory reflux, of which 29% had reflux symptoms [17]. Han et al. evaluated 997 children who underwent adenoidectomy and found that 81 children had gastroesophageal reflux and 8 had postoperative complications. They believed that reflux was a risk factor for complications after adenoidectomy [18]. Moreover, another study has highlighted that the increased respiratory movement of OSA in children (mainly due to adenoid hypertrophy) produces tremendous negative pressure, which induces reflux, then produces inflammation and sensory abnormalities in the throat, leading to the aggravation of OSA [19].

RSI and RFS are the simplest clinical screening methods for diagnosing pharyngeal reflux [20]. The diagnostic value of this scale for adult pharyngeal reflux has been recognized [21]. However, their diagnostic value in children with pharyngeal reflux remains largely unknown. In this study, the positive adenoid pepsin in children was used as the gold standard to evaluate the correlation and accuracy of RSI and RFS in predicting adenoid hypertrophy and pharyngeal reflux. We found that RSI and RFS scales, whether applied alone or in combination, have low sensitivity in diagnosing pharyngeal reflux in children with adenoidal hypertrophy, suggesting that these two scales are unsuitable for the preliminary screening of children with adenoidal hypertrophy in the diagnosis of pharyngeal reflux. In RSI, we found that most children with adenoidal hypertrophy had excessive sputum or nasal regurgitation (55.8%), annoying cough (53.5%), and cough after eating or lying down (35.9%). In RFS, we found that most children with adenoidal hypertrophy had mucus attachment in the larynx (53.5%), partial or complete disappearance of the laryngeal chamber (23.3%), and vocal cord edema (27.9%).

Gastric reflux contains gastric acid, pepsin, bile, trypsin, and other components [22]. Among them, pepsin is an essential proteolytic enzyme in the digestive system, and it is also the main invasive component in pharyngeal reflux [23]. In this study, pepsin was detected in hypertrophic adenoids, and the positive rate was as high as 69.77%, suggesting that pharyngeal reflux widely existed in children with hypertrophic adenoids. At the same time, the higher the grade of adenoid hypertrophy, the higher the expression intensity of pepsin (*r* = 0.576, *P* < 0.01), indicating that pepsin plays an essential role in developing adenoid hypertrophy.

In conclusion, we found that RSI and RFS scales have low sensitivity and poor diagnostic value for AH children and are unsuitable for diagnosing pharyngeal reflux in children with AH. In addition, AH in children is correlated with pathological reflux of gastric contents such as pepsin. Pathological reflux of gastric contents to the nasopharynx might be one of the causes of adenoid hypertrophy.

## Figures and Tables

**Table 1:** RSI scale.

Symptom	Cases	Proportion (%)
Hoarseness or dysphonia	5	11.6
Excessive sputum or nasal mucus reflux	24	55.8
Cough after eating or lying down	17	35.9
Dyspnea or recurrent asphyxia	4	9.3
Foreign body sensation in the throat	25	58.1
Heartburn, chest pain, stomach pain	2	4.7
Bad for swallowing food, water, or tablets	10	23.3
Annoying cough	23	53.5
Throat clearing	25	58.1

**Table 2:** RFS scales (electronic laryngoscopy of children).

Laryngoscopic appearance	Cases	Proportion (%)
Pseudovocal groove exists	5	11.6
Partial or complete disappearance of laryngeal chamber	10	23.3
Diffuse erythema of laryngeal cavity	6	13.9
Edema of the vocal cord	12	27.9
Mucus attachment in the larynx	23	53.5
Diffuse laryngeal edema	6	13.9
Thickening of posterior commissural mucosa	7	16.3
Granuloma	0	0

**Table 3:** Comparison between adenoid hypertrophy grading and pepsin expression.

Pepsin expression	*Adenoidal hypertrophy grading*			
	Level 1	Level 2	Level 3	Level 4
−	—	—	9	4
+	—	—	6	3
++	—	—	5	8
+++	—	—	2	5

*χ * ^2^	7.68			
*r*	0.576			
*P*	0.003			

**Table 4:** Sensitivity and specificity of RSI, RFS, and RSI combined with RFS in diagnosing pharyngeal reflux.

	Pepsin (+)	Pepsin (−)
RSI (+)	3	1
RSI (−)	25	9
RFS (+)	18	8
RFS (−)	16	5
RSI + RFS (+)	20	9
RSI + RFS (−)	15	7

RSI: reflux symptom index; RFS: reflux finding score.

**Table 5:** Patients characteristics.

	Total (*n* = 43)	LPR (+) (*n* = 8)	LPR (−) (*n* = 35)	*P*
Age	6.73 ± 3.08	6.91 ± 3.04	6.52 ± 2.82	0.51
*Sex (no, %)*				
Female	20 (46.4%)	5 (62.5%)	20 (57.1%)	0.39
Male	23 (53.5%)	3 (37.5%)	15 (42.9%)	
LPR (median)	20	49	27	0.70
Acid regurgitation (median)	12	46	20	0.06
Mouth breathing (no, %)	34 (79.1%)	5 (62.5%)	29 (82.9%)	0.70
Snoring (no, %)	28 (65.1%)	4 (50%)	24 (68.6%)	0.34
Stoppage of breathing (no, %)	3 (6.9)	1 (1.3%)	2 (5.7%)	0.40
URTI (no, %)	4 (9.3%)	2 (25%)	2 (5.7%)	0.28
Heartburn (no, %)	2 (4.7%)	1 (1.3%)	1 (2.9%)	0.08

URTI: upper respiratory tract infection; LPR: laryngopharyngeal reflux.

## Data Availability

The labeled dataset used to support the findings of this study are available from the corresponding author upon request.
